# The Real Code of Leonardo da Vinci

**DOI:** 10.2174/157340308783565401

**Published:** 2008-02

**Authors:** Leiv Ose

**Affiliations:** Lipid Clinic, Medical Department, Rikshospitalet HF, NO 0027 Oslo Norway

**Keywords:** Familial hypercholesterolemia; Leonardo da Vinci.

## Abstract

Leonardo da Vinci was born in Italy. Among the researchers and scientists, he is favourably known for his remarkable efforts in scientific work. His investigations of atherosclerosis judiciously combine three separate fields of research. In 1506, he finished his masterpiece, painting of Mona Lisa. A careful clinical examination of the famous painting reveals a yellow irregular leather-like spot at the inner end of the left upper eyelid and a soft bumpy well-defined swelling of the dorsum of the right hand beneath the index finger about 3 cm long. This is probably the first case of familial hypercholesterolemia (FH). The FH code of Leonardo da Vinci was given immense consideration by scientists like Carl Muller, who described the xanthomas tuberosum and angina pectoris. On the contrary, Akira Endo searched for microbial metabolites that would inhibit HMG-CoA reductase, the rate-limiting enzyme in the synthesis of cholesterol and finally, Michael Brown and Joseph Goldstein published a remarkable series of elegant and insightful papers in the 70s and 80s. They established that the cellular uptake of low-density lipoprotein (LDL) essentially requires the LDL receptor. In conclusion: this was the real Code of Leonardo da Vinci.

## INTRODUCTION

Leonardo da Vinci was a famous Italian artist, inventor and scientist born in Italy in 1452 and died in France in 1519 at the age of 67. Leonardo da Vinci’s description of atherosclerosis is well known [1]. His scientific work is most remarkable in his multilateral approach to the problem. His investigations of atherosclerosis are no exception, which present a clear-cut description by combining three separate fields of research. Firstly, his study of hydrodynamics paved the way to bring to light various factors governing the flow of fluid. Secondly, the study of anatomy delineated his observations on the effects of age on anatomical structures, particularly on blood vessels. Thirdly, his concept of nutrition, added a final touch to his description of the process. The thickening of the arterial wall pointed out the difference between the vessels in the young and in the old. As the vessels become old, their branches loose their structure and become much more tortuous, resulting in thickening of the walls. Leonardo, however, noticed that it is not the thickening of the blood that hampers flow through the vessels, as the blood in the vessels does not thicken, because the old blood is continuously replaced with the new blood.

**Fig. (1):**
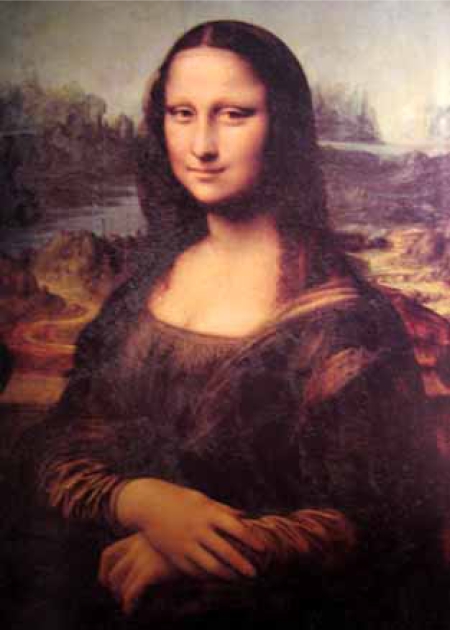
Madonna Lisa Maria de Gherardini (Mona Lisa) 1479-1516 painted by Leonardo da Vinci in 1503-1506. The painting is in Louvre, Paris.

**Fig. (2):**
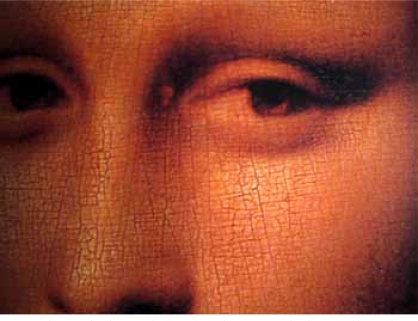
The painting of Mona Lisa revealed a yellow irregular leather-like spot at the inner end of the left upper eyelid.

**Fig. (3):**
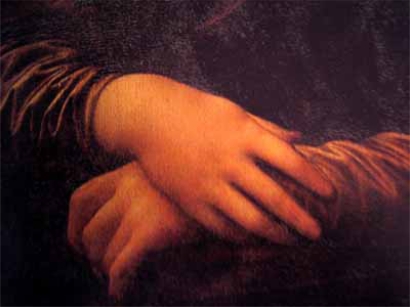
A soft bumpy well-defined swelling is revealed on the dorsum of the right hand beneath the index finger being about 3 cm long.

In 1503, Leonardo started the painting of Madonna Lisa Maria de Gherardini born in Florence in 1479 and died at the age of 37 (Fig.**1**). He worked on the painting for 4 years. A careful clinical examination of the famous painting reveals a yellow irregular leather-like spot at the inner end of the left upper eyelid (Fig. **2**) and a soft bumpy well-defined swelling of the dorsum of the right hand beneath the index finger about 3 cm long (Fig. **3**). An infrared detailed photograph published in 1974 reveals that the yellow skin alteration was an integral part of the painting at the time of its initiation [2]. The yellow spot looked somewhat similar to what later came to be known as xanthelasma. These skin lesions are often observed in people suffering from inherited forms of hyperlipidemia.

**Fig. (4):**
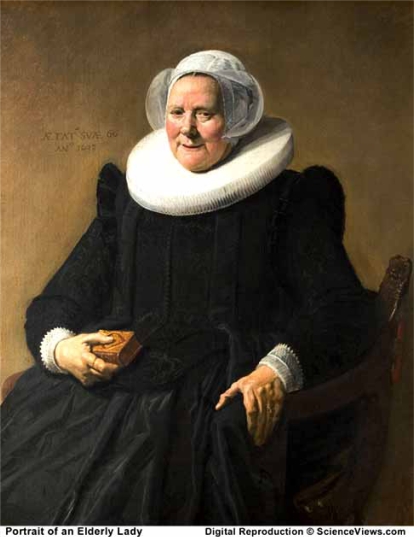
*Portrait of an Elderly Lady* painted in 1633 by Frans Hals 1580-1 - 1666. The painting is in the National Gallery of Art in Washington DC.

**Fig. (5):**
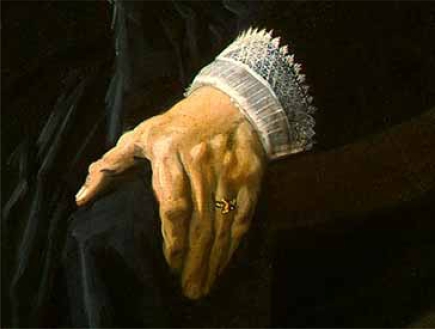
The *Portrait of an Elderly Lady* showed the classic lesions of xanthomas on the dorsum of the hand of a 60-year old lady.

Visual arts, particularly of the Flemish School, in combination with historical documentation have been an important tool for learning observational acuity in rheumatology and various skin disorders. An observation of xanthelasma in the eye region in association with subcutaneous lipoma of the dorsum of the hand in women between the age of 25 and 40 may indicate early diagnosis of the metabolic disorder - hyperlipidemia - that may later be described as being familiar. The stronger evidence is supposed to be the observation of a corneal arcus and it was however not found in this portrait. The occurrence of xanthelasma lipoma in a women aged 25-30 is probably not coincidental. Mona Lisa died at the age of 37, but the cause of death could not be figured out. In conclusion, this portrait painted in 1506 is probably the first evidence of familial hypercholesterolemia (FH), which appeared long before a later description in 1852 by Addison and Gall. Frans Hals, the 17th century Dutch painter, again painted the xanthomas in an attempt to further elucidate the clinical disorder. This *Portrait of an Elderly Lady* of 1663, showed the classic lesions on the dorsum of the hand of a 60-year old, who probably had heterozygous FH (Fig. **4**, Fig. **5**). The painting did not depict any traces of a history of manifestation of xanthelasma in that woman [3].

In 1873, Fagge described a case of xanthomastosis with cardiovascular symptoms and in 1889, Lehzen and Knaus reported the case of an 11-year-old girl presenting with the steadily growing xanthomas, accompanied by cardiovascular symptoms. She died suddenly and the post-mortem study revealed exanthomatous deposits in the aorta, with the narrowing isthmus, as in other large arteries. Later, her sister manifested similar skin changes, highlighting this case to be the first report on homozygous FH [4].

Francis Harbitz from Norway published several papers from 1920 to 1927 elucidating on the tumours of tendons sheets, joint capsules and ultimately the occurrence of multiple xanthomas, and sudden death. In all publications so far regarding hereditary xanthomatosis associated with heart disease, the authors did not pay significant attention to the condition due to being manifested infrequently [5].

In 1937, almost 70 years ago, Carl Müller, identified the first patient ever who presented with xanthomas tuberosum and angina pectoris. Müller made a preliminary report of a number of cases in which he expressed that hypercholesterolemia is a frequent and important factor in heart disease, following which several new patients were referred to Müller and in 1939 he published his landmark paper [6]. He then reported on 17 families with 76 family members out of which 68 manifested symptoms of possible heart disease. Half of the patients died suddenly and xanthomas were noted alone or in combination with xanthelasma. Serum cholesterol level varied from 4 to 15 mmol/l. However, no correlation between levels of serum cholesterol and xanthomas or xanthelasma was observed. Müller noted that in xanthomatosis, the vascular changes frequently manifested themselves in coronary disease and angina pectoris. In such a systemic disease one would expect other localizations of symptoms – however, he only came across a single case of death from stroke. He concluded his paper with the following statement: "It has been shown that the cholesterol content of the blood can be reduced by a diet poor/low in cholesterol. I have ordered a diet with no yolk of eggs, butter, cream, fat milk or animal fat and thyroid tablets. I have not been able to formulate any opinion in regard of the effects. The treatment may be of prophylactic value to persons with hereditary predisposition".

In 1971, Akira Endo started a project to search the microbial metabolites that would inhibit HMG-CoA reductase, the rate-limiting enzyme in the synthesis of cholesterol, with the intent that the suppression of the de novo cholesterol synthesis in the body by inhibiting HMG-CoA reductase would reduce plasma cholesterol in humans. Over a 2-year period, about 6000 microbial strains were tested. A strain of *Penicillum citrinum* was found to produce active compounds and mevastatin was found [7]. Later, analogues of mevastatin were also found such as: lovastatin, simvastatin and pravastatin.

A remarkable series of elegant and insightful papers was published by Brown and Goldstein in the 70s and 80s. They established that the cellular uptake of low density lipoprotein (LDL) essentially requires the LDL receptor. In the complete absence of the LDL receptor, the LDL concentration in plasma can build up to 20-25 mmol/l. This discovery of the nature of the defective gene in familial hypercholesterolemia by Brown and Goldstein turned out to be a major milestone in the lipoprotein field and they were awarded the Nobel Price in Physiology and Medicine in 1985 [8]. The other forms of autosomal dominant hypercholesterolemia are familial defective apoB-100 which is caused by mutations in the apoB gene [9] and FH3, which is caused by mutations in the proprotein convertase subtilisin/kexin type 9 (PCK9) gene [10]. In addition to the dominant hypercholesterolemia, there is one autosomal recessive form caused by mutations in the LDL receptor adaptor protein [11].

The real Code of da Vinci - is the code for FH. The first case was presented 500 years ago by Leonardo da Vinci in his painting of Mona Lisa and its solution was found through the work of scientists like Müller, Endo, Brown and Goldstein.
